# Supplementary material for: Subtype-specific neutralizing antibodies promote antigenic shift during influenza virus co-infection
Source: Virulence. 2026 Jul 21;17(1):2707716. doi: 10.1080/21505594.2026.2707716 (PMC13418698; doi:10.1080/21505594.2026.2707716)
Supplement: The approval protocol submitted to the institutional review board.pdf [file KVIR_A_2707716_SM2798.pdf]

内蒙古大学生命科学学院动物实验伦理审查表

Application Format for Ethical Approval for Research Involving Animals of Laboratory Animal Platform, Inner Mongolia University

申请日期： 2020 年 03 月 03 日　　　　批准文号： IMU-mouse-2020-048
Appl. Date： 2020 Y 03 M 03 D　　　　IACUC Issue No. IMU-mouse-2020-048

<table>
<tr><td colspan="4">申请单位： 内蒙古大学
Department： Inner Mongolia University</td></tr>
<tr><td colspan="2">申请人姓名(Name of applicant) 王国俊

电话/Tel. No.: 13081522302
邮箱/Email： guojun.wang@imu.edu.cn</td><td colspan="2">课题负责(Name of Principal Investigator) 王国俊

电话/Tel. No.: 13081522302
邮箱/Email： guojun.wang@imu.edu.cn</td></tr>
<tr><td colspan="4">课题名称 ： 毒株特异性中和抗体在流感病毒共感染促进流感病毒的抗原转变
Program title: Subtype-specific neutralizing antibodies promote antigenic shift during influenza virus co-infection</td></tr>
<tr><td colspan="4">课题来源：国家自然科学基金项目
Sponsor： Regional Project of the National Natural Science Foundation of China</td></tr>
<tr><td rowspan="7">拟使用动物信息
Animal to be used</td><td colspan="3">实验动物使用许可证号 SYXK（蒙）2020-0006
Name and certificate number of the facility SYXK 2020-0006</td></tr>
<tr><td colspan="3">动物来源 北京斯贝福（北京）生物技术有限公司
Animal origin SPF (Beijing) Biotechnology Co., Ltd
动物来源单位生产许可证号 SCXK（京）2019-0010
Certificate number SCXK2019-0010
质量合格证 ☑ 有 ☐ 无
Certification of fitness ☑ Y ☐ N</td></tr>
<tr><td>品种/品系（breed/strain）：小鼠/BALB/c</td><td>等级（Grade）： SPF</td><td>数量（Number）：
（♀200 只； ♂0 只）</td></tr>
<tr><td>体重（Weight）： 18-20g</td><td>月龄（Age）： 1.5-2 month</td><td rowspan="2">其他（Others）</td></tr>
<tr><td>参与动物实验的人数(Number of Implement)： 2</td><td>经专业培训的人数(Number of certificate)： 2</td></tr>
<tr><td colspan="3">开始日期（Entering date）: 2020 年 6 月 1 日</td></tr>
<tr><td colspan="3">结束日期（Ending date）: 2024 年 12 月 31 日</td></tr>
</table>

拟开展动物实验的详细信息 Detailed information of the experiments on animals	1. 实验要点，包括实验目的、必要性、实验方法、观测指标、动物处死方法<br>Outline of experiments（experimental objective, necessity, experimental methods, observation target, death conduct）<br>①实验目的：建立荧光流感病毒感染小鼠模型，明确中和抗体在体内对流感病毒重组的影响。<br>②必要性：流感病毒具有高度传染性和致病性，可引发季节性流行及全球性大流行，对人类健康和公共卫生安全构成严重威胁。小鼠作为常用的模式动物，其生理机制与人类具有一定相似性，且BALB/c 小鼠对流感病毒敏感性高、遗传背景稳定，是研究流感病毒感染机制、致病性及防控策略的理想模型。本实验通过构建荧光流感病毒感染小鼠模型，能够直观观察病毒感染后的生理变化（体重、存活情况），为解析不同类型中和抗体对流感病毒重组的影响提供关键的体内实验数据，具有重要的科研价值和现实意义。<br>③实验方法：1、实验动物：选用 6~8 周龄 SPF 级 BALB/c 雌性小鼠。2、麻醉处理：实验组小鼠通过腹腔注射戊巴比妥进行麻醉。3、病毒接种：待小鼠麻醉后，采用经鼻接种方式进行病毒感染，使用移液器向每只小鼠鼻腔内滴入 50 μL 的病毒液。4、实验分组：实验设置多个不同滴度的流感病毒组以及在感染前给予特定抗体处理的抗体干预组，每组均包含不少于 5 只小鼠，以保证实验结果的统计学意义。5、观察周期：对所有实验组和对照组小鼠进行连续 14 天的观察记录。<br>④观测指标：1、体重变化：每日固定时间称量并记录每只小鼠的体重，计算体重相对于初始体重的变化率，以此评估病毒感染对小鼠身体状况的影响。2、存活率：每日观察并记录各组小鼠的存活情况，统计 14 天观察期内的小鼠存活率，分析不同实验条件下病毒感染的致死性差异。当小鼠体重下降至小于初始体重的 25%时，视为达到实验终点，进行安乐死处理。<br>⑤动物处死方法：采用过量麻醉注射法，通过注射超剂量麻醉剂，抑制中枢神经系统至呼吸、心跳停止，符合安乐死规定范畴，痛苦程度极低。<br>① Experimental Objective: To establish a mouse model of influenza virus infection with fluorescent labeling, and to clarify the effect of neutralizing antibodies on influenza virus reassortment in vivo.<br>② Necessity: Influenza virus is highly contagious and pathogenic, capable of causing seasonal epidemics and global pandemics, posing a serious threat to human health and public health security. Mice, as commonly used model animals, share certain physiological similarities with humans. In particular, BALB/c mice exhibit high susceptibility to influenza virus and have a stable genetic background, making them an ideal model for studying the mechanisms of influenza virus infection, pathogenicity, and prevention strategies. By constructing a fluorescent influenza virus-infected mouse model, this experiment allows direct observation of physiological changes (body weight, survival) following viral infection, providing critical in vivo experimental data for analyzing the impact of different types of neutralizing antibodies on influenza virus reassortment. This research holds significant scientific value and practical relevance.<br>③ Experimental Methods: 1、Experimental animals: SPF-grade female BALB/c mice aged 6-8 weeks are selected. 2、Anesthesia: Mice in the experimental groups are anesthetized via intraperitoneal injection of Pentobarbital. 3、 Virus inoculation: After anesthesia, viral infection is performed via intranasal inoculation. Using a pipette, 50 μL of virus solution is instilled into the nasal cavity of each mouse. 4、Experimental grouping: The experiment includes multiple groups with different influenza virus titers, as well as an antibody intervention group in which specific antibodies are administered prior to infection. Each group contains no fewer than 5 mice to ensure statistical significance of the results. 5、Observation period: All experimental and control groups are observed and recorded continuously for 14 days.<br>④ Observation Indicators: 1、Body weight change: The body weight of each mouse is measured and recorded at a fixed time daily. The rate of change relative to the initial body weight is calculated to assess the impact of viral infection on the mice's physical condition. 2、Survival rate: The survival status of mice in each group is observed and recorded daily. The survival rate over the 14-day observation period is calculated to analyze differences in lethality under various experimental conditions. When a mouse's body weight drops below 25% of its initial weight, it is considered to have reached the experimental endpoint and is euthanized.<br>⑤ Animal Euthanasia Method: Overdose anesthesia injection is used. An excessive dose of anesthetic is administered to suppress the central nervous system until respiration and heartbeat cease. This method

complies with euthanasia regulations and causes minimal suffering.

2. 选择实验动物种类和数量的原因(Reasons for the choice of species and numbers of animals to be used).

选择实验动物种类原因：（1）BALB/c 小鼠为近交系小鼠（近交系数 ＞99%），个体间遗传背景高度均一，基因同源性高，无遗传杂合性导致的个体差异。可最大限度减少遗传因素干扰实验结果；（2）BALB/c 小鼠对如流感病毒等特定病原体的敏感性和反应模式明确，可精准研究病原体感染机制；（3）BALB/c 小鼠免疫应答稳定，可减少因动物免疫状态差异导致的实验误差，避免无效实验（4）且 BALB/c 小鼠繁殖周期稳定，易获得充足的实验个体，因此选择 BALB/c 小鼠作为实验动物。

选择数量原因：

实验分组：

（1）PR8、PR8-NSmCherry、VNH5N1 和 VN-NSGFP 感染 BALB/c 小鼠模型计算病毒 MLD_{50}

将病毒分别稀释 10^1-10^6PFU 不同浓度梯度，共 6 组，每组 5 只小鼠，PBS 为空白对照组，共 125 只

（2）PR8-NSmCherry 和 VN-NSGFP 在体内稳定性检测

PR8-mCherry 和 VN-GFP 病毒用无菌 PBS 稀释至 10^4PFU/50μL。采用戊巴比妥进行麻醉注射小鼠，待小鼠麻醉后，用移液器经鼻滴注 50 μL 病毒液。并需采集上一代小鼠肺组织进行匀浆后滴鼻至新的小鼠中，连续传三代。共 2 组，每组 15 只小鼠，PBS 为空白对照组 5 只，共 35 只小鼠。

（3）抗体皮下注射小鼠后 PR8-NSmCherry 和 VN-NSGFP 混合液感染小鼠

将 PBS、6F12、GG3 和 1H4 三种抗体经皮下注射小鼠后，2 小时后病毒滴鼻感染小鼠(每组 10 只)，其中每组在感染病毒 3 天后需采集 5 只小鼠肺组织进行病毒滴度及子代病毒基因型的鉴定实验，剩余 5 只小鼠连续观察 14 天小鼠体重和生存率，共需 40 只小鼠。

因此，本研究共需 200 只小鼠。

Reasons for selecting the experimental animal species:

(1) BALB/c mice are an inbred strain (inbreeding coefficient ＞ 99%), with highly uniform genetic backgrounds among individuals and high genetic homology, eliminating individual differences caused by genetic heterozygosity. This minimizes interference from genetic factors on experimental results.

(2) BALB/c mice have well-defined susceptibility and response patterns to specific pathogens such as influenza virus, enabling precise study of pathogen infection mechanisms.

(3) BALB/c mice exhibit stable immune responses, reducing experimental errors caused by differences in immune status among animals and avoiding ineffective experiments.

(4) BALB/c mice have a stable reproductive cycle, making it easy to obtain sufficient experimental individuals. Therefore, BALB/c mice were selected as the experimental animals.

Reasons for selecting the number of animals:

Experimental groups:

(1) Calculation of viral MLD_{50} in BALB/c mice infected with PR8, PR8-NSmCherry, VNH5N1, and VN-NSGFP

The viruses were diluted into different concentration gradients from 10^1 to 10^6 PFU, with 6 groups in total, each containing 5 mice. PBS was used as the blank control group. Total: 125 mice.

(2) Stability testing of PR8-NSmCherry and VN-NSGFP *in vivo*

PR8-mCherry and VN-GFP viruses were diluted to 10^4 PFU/50 μL with sterile PBS. Mice were anesthetized by intraperitoneal injection of pentobarbital. After anesthesia, 50 μL of virus solution was instilled intranasally using a pipette. Lung tissues from the previous generation of mice were collected, homogenized, and then instilled intranasally into new mice, with continuous passage for three generations. A total of 2 groups, each with 15 mice, plus 5 mice in the PBS blank control group. Total: 35 mice.

(3) Infection of mice with a mixture of PR8-NSmCherry and VN-NSGFP after subcutaneous antibody injection: PBS, 6F12, GG3, and 1H4 antibodies were injected subcutaneously into mice. Two hours later, the mice were infected intranasally with the virus (10 mice per group). Three days after infection, lung tissues were collected from 5 mice in each group for viral titer determination and progeny virus genotyping. The remaining 5 mice in each group were observed continuously for 14 days for body weight and survival rate. Total: 40 mice.

Therefore, a total of 200 mice are required for this study.

3. 详细列出对动物可能造成的所有可预期的伤害，包括动物运输、动物饲养方式、实验操作等可能产生伤害或不适的细节以及 Description of the overall harms expected to be experienced by the animals-including details of the likely adverse effects of each protocol, cage breeding and the steps which will be taken to control these adverse effects.

对动物可能造成的所有可预期的伤害<br>All foreseeable harm to animals		拟采取的防控措施<br>Proposed prevention and control measures
动物运输<br>Animal transport	环境应激：运输箱内温度波动、湿度不适、通风不良导致缺氧或闷热；<br>Environmental stress: Temperature fluctuations, inappropriate humidity, poor ventilation leading to hypoxia or heat stress inside the transport box.	运输箱采用带通风孔材质，避免过度拥挤，箱内铺垫无菌吸尿垫；运输前检查箱内温度（维持20-26℃）、湿度（40%-60%），全程实时监控并记录环境参数；运输时间控制在 8 小时内。<br>Transport boxes are made of ventilated material to avoid overcrowding, and lined with sterile absorbent pads; before transport, check the temperature (maintain 20 – 26 ° C) and humidity (40% – 60%) inside the box, and monitor and record environmental parameters in real time throughout the journey; transport time should be controlled within 8 hours.
	物理颠簸与惊吓：运输过程中急刹、震动导致小鼠碰撞受伤；陌生环境引发应激反应<br>Physical jolts and fright: Sudden braking or vibration during transport causing collision injuries; unfamiliar environment inducing stress response.	运输车辆匀速行驶，避免急加速/急刹车；运输箱外包裹遮光布减少光线刺激，保持环境安静；抵达实验室后，将小鼠置于饲养间适应环境 48 小时（不进行任何操作），观察饮食、排便情况。<br>Drive the transport vehicle at a constant speed, avoiding sudden acceleration or braking; cover the transport box with a light-blocking cloth to reduce light stimulation; keep the environment quiet; upon arrival at the laboratory, allow mice to acclimate in the housing room for 48 hours (no procedures performed) and observe food intake and defecation.
	饲养间温度/湿度异常、光照紊乱、噪音过大导致小鼠应激。<br>Temperature/humidity abnormalities, light cycle disruption, excessive noise in	饲养间设置温度维持 22±2℃，湿度 45%-55%，每日记录环境参数；光照采用 12h 光/12h 暗循环（早 7 点-晚 7 点光照），避免强光直射笼具；饲养间保持安静（噪

	动物饲养方式<br>Animal housing	the housing room causing stress in mice.	音≤60 分贝），禁止在饲养区大声交谈或使用震动设备；每周更换笼具、垫料（使用无菌玉米芯垫料，避免粉尘刺激），每日清理食盒、水瓶确保无菌饮水和饲料充足。<br>The housing room temperature is maintained at 22±2° C, humidity at 45% - 55%, with environmental parameters recorded daily; lighting follows a 12-hour light/12-hour dark cycle (lights on from 7:00 AM to 7:00 PM), avoiding direct bright light on cages; the housing room is kept quiet (noise ≤ 60 dB), and loud conversations or use of vibrating equipment are prohibited in the housing area; cages and bedding are changed weekly (using sterile corncob bedding to avoid dust irritation), and food hoppers and water bottles are cleaned daily to ensure sterile drinking water and sufficient feed.
		同笼小鼠因领地争夺发生打斗，导致皮肤咬伤、抓伤。<br>Fighting among cage mates due to territorial disputes, resulting in skin bites or scratches.	同笼小鼠为同窝、同性别，每笼饲养数量≤5 只 BALB/c 小鼠；每日观察小鼠体表是否有伤口，发现打斗迹象立即分笼。<br>House mice from the same litter and same sex; ≤5 BALB/c mice per cage; inspect mice daily for wounds on the body surface; separate immediately if signs of fighting are observed.
	实验操作<br>Experimental procedures	保定应激：实验操作时小鼠因被抓握产生恐惧，导致挣扎、心率加快、血压升高。<br>Restraint stress: Fear caused by handling during procedures, leading to struggling, increased heart rate, and elevated blood pressure.	操作人员需经专业培训，采用轻柔抓握，右手拇指、食指捏住小鼠耳后颈部皮肤，左手托住腹部，避免用力挤压；单只小鼠操作时间控制在 5 分钟内，避免长时间保定。<br>Operators must undergo professional training and use gentle handling: grasp the loose skin at the back of the mouse's neck between the thumb and index finger of the right hand, support the abdomen with the left hand, and avoid excessive squeezing; the

			handling time for a single mouse should be controlled within 5 minutes to avoid prolonged restraint.
		注射相关伤害：皮下注射时操作不当导致出血、脏器损伤等<br>Injection-related injury: Improper technique during subcutaneous injection causing bleeding or organ damage.	选择腹股沟皮下疏松组织进针，针头斜面朝上，进针角度 15° 至 30°，回抽无回血后缓慢推注药液，避免将药液注入肌肉或腹腔；所有注射针头使用一次性无菌针头；注射前对局部皮肤用 75%酒精消毒。<br>Choose loose subcutaneous tissue on the nape of the neck or groin area for injection; insert the needle with the bevel facing upward at a 15° to 30° angle; aspirate to confirm no blood return before slowly injecting the solution; avoid injecting into muscle or abdominal cavity; use sterile disposable needles for all injections; disinfect the local skin with 75% alcohol before injection.
	4. 是否使用有毒（害）物质（放射、化学毒、其他）<br>Poisonous (harmful) material (radiate, chemical poison and other) being used<br>否 No		

声明

1. 我将自觉遵守实验动物福利伦理相关法规和各项规定，同意接受伦理委员会和实验动物管理者的监督与检查。2. 本人保证本申请表中所填内容真实、详尽和易懂。

Declaration:

1. I will abide by the law and regulation stipulation, and accept the supervision and inspection by the committee and laboratory animal department. 2. The information I have giver is accurate, detailed and comprehensive.

声明人（Declarant）：王国俊

课题负责人签名（Signature of PI ）：[illegible]

实验执行人签名 （Signature of Experiment Investigator）：刘梦楠

审查依据 Inspection contents	1. 该项目是否必须用实验动物进行实验？☑ 是 ☐ 否<br>能否用计算机模拟、细胞培养等非生命方法替代动物或用低等动物替代高等动物进行实验？ 是 ☐ ☑ 否<br>Does laboratory animal must be used in the project? ☑ Yes ☐ No<br>Could other methods such as computer simulation ,cell cultivation or using the low-grade animal instead of the high-grade animal? ☐ Yes ☑ No<br><br>2. 项目所用动物的品种品系、质量等级、规格是否合适？☑ 是 ☐ 否<br>能否通过改良设计方案或用高质量的动物来减少所用动物的数量？ ☐ 是 ☑ 否<br>Are animal strain, grade and specifications of animals suitable? ☑ Yes ☐ No<br>Could the quantity of animals be reduced by improving the study design or using high quality animals? ☐ Yes ☑ No<br><br>3. 能否通过改进实验方法、调整实验观测指标、改良处死动物的方法来优化实验方案，善待动物？<br>☐ 是 ☑ 否<br>Could the study design and animal treatment be refined by ameliorating experimental method, adjusting observational index, executing animal method? ☐ Yes ☑ No
审查结果 Results of inspection	内蒙古大学生物伦理委员会意见<br>Approval opinion of Ethics Committee<br><br>☑ 同意 ☐ 修改后同意 ☐ 不同意<br>☑（Agree） ☐ （Agree after revised） ☐ （Disagree）

内蒙古大学生物伦理委员会负责人签名

Signature of Chairman of Ethics Committee

胡[illegible]

年 月 日

申报说明：申报时，请提交本表一式两份及电子版。

Notice: Submitting the Application Format in duplicate and an electronic edition.
