# Supplementary material for: Subtype-specific neutralizing antibodies promote antigenic shift during influenza virus co-infection
Source: Virulence. 2026 Jul 21;17(1):2707716. doi: 10.1080/21505594.2026.2707716 (PMC13418698; doi:10.1080/21505594.2026.2707716)
Supplement: Supplemental material table S1.docx [file KVIR_A_2707716_SM2797.docx]

Supplemental material

Table S1 Primers used to generate amplicons for high-resolution melt analysis

| Segment | Forward primer | Reverse primer |
| --- | --- | --- |
| PB2 | 477F  CCATGCAGATCTCAGTGCTAAA | 1584R  TTTCTCTGTTCCCTGGGTTTC |
| PB1 | 2016F  TCTTCCCCAGCAGTTCATA | 2319R  AGTAGAAACAAGGCATTTTTTCA |
| PA | 1864F  AACAAATCAGAAACATGGCC | 2077R  TCAATTGCTTCATATAGCCC |
| HA | 1584F  GGAGTRAAATTGGAATCAAT | 1755R  AGTAGAAACAAGGGTGTTTTT |
| NP | 1213F  CCAGAAGYGGAGGAAACACC | 1544R  AGTAGAAACAAGGGTATTTTTC |
| NA | 1167F  GATTGGTCAGGRTATAGCGG | 1389R  AGTAGAAACAAGGAGTTTTTTGAAC |
| M | 159F  GGCTAAAGACAAGACCAATCCT | 574R  CTCCATAGCCTTAGCTGTAGTG |
| NS | 1626F  CAGAAGTTTGAAGAAATAAG | 1756R  AGTAGAAACAAGGGTGTTTTTTATC |
