# Supplementary material for: Subtype-specific neutralizing antibodies promote antigenic shift during influenza virus co-infection
Source: Virulence. 2026 Jul 21;17(1):2707716. doi: 10.1080/21505594.2026.2707716 (PMC13418698; doi:10.1080/21505594.2026.2707716)
Supplement: Table1.docx [file KVIR_A_2707716_SM2795.docx]

Table 1 Characterization of antibodies employed in this study

| Name | Source | Specificity | Reactivity to H1 virus? | Reactivity to H5 virus? |
| --- | --- | --- | --- | --- |
| 6F12 | Mouse | HA Stalk | Yes | No |
| GG3 | Mouse | HA Stalk | Yes | Yes |
| 1H4 | Mouse | HA Head | No | Yes |
